# A Generalized Approach to Solving Deep Learning‐Based Quantitative Susceptibility Mapping and Quantitative Blood Oxygen Level Dependent Magnitude (QSM + qBOLD or QQ) for Oxygen Extraction Fraction (OEF) Mapping Across Diverse Acquisition Schemes

**DOI:** 10.1002/mrm.70524

**Published:** 2026-08-04

**Authors:** Tian Qiu, Ada Ally, Arpita Misra, Gloria C. Chiang, Thanh D. Nguyen, Susan A. Gauthier, Shun Zhang, Yi Wang, Junghun Cho

**Affiliations:** ^1^ Department of Biomedical Engineering George Washington University Washington DC USA; ^2^ Department of Biomedical Engineering State University of New York at Buffalo Buffalo New York USA; ^3^ Department of Radiology Weill Cornell Medicine New York New York USA; ^4^ Department of Radiology Tongji Hospital, Tongji Medical College, Huazhong University of Science and Technology Wuhan Hubei China

**Keywords:** deep learning, oxygen extraction fraction, QQ, quantitative blood level dependent imaging, quantitative susceptibility mapping

## Abstract

**Purpose:**

QQ, a recently proposed oxygen extraction fraction (OEF) mapping technique combining quantitative susceptibility mapping (QSM) and quantitative blood oxygen level‐dependent (qBOLD) (QSM + qBOLD = QQ), generates OEF maps noninvasively from a single routine MRI sequence, without requiring vascular challenges used in other OEF approaches. A deep learning approach, QQ‐NET, further enables rapid 3D OEF reconstruction (˜1.5 min), but it is trained on a fixed echo‐time (TE) scheme and must be retrained whenever acquisition protocols differ, limiting its clinical applicability. This study introduces QQ‐F, a novel deep learning approach designed to eliminate the need for retraining.

**Methods:**

QQ‐F incorporates a feature extraction unit that derives QQ model‐related features as inputs, rather than relying directly on raw signals. For a fair comparison, QQ‐F was trained using the same 3D multi‐echo gradient echo (mGRE) dataset as QQ‐NET, acquired from 26 ischemic stroke patients. Both models were tested using simulations and data from 24 multiple sclerosis (MS) and 30 dementia patients acquired with varying TE sequences.

**Results:**

In simulations, QQ‐F provided more accurate OEF maps than QQ‐NET with lower mean absolute error. In patient datasets–particularly dementia datasets, where TE values differed substantially from QQ‐NET's training protocol—QQ‐F yielded significantly higher lesion‐to‐normal tissue contrast than QQ‐NET, indicating superior robustness to acquisition variability.

**Conclusion:**

QQ‐F enables deep learning‐based QQ OEF mapping across diverse MR acquisition protocols without retraining, thereby enhancing the clinical scalability of QQ‐based OEF mapping.

## 1 Introduction

Quantitative mapping of oxygen extraction fraction (OEF) is critical for evaluating brain tissue viability and function in neurologic disorders [1, 2, 3, 4]. Although ^15^O‐PET is the reference standard, its clinical use is limited by the short half‐life of ^15^O (˜2 min), requiring an on‐site cyclotron. Given MRI's wide availability, MRI‐based OEF mapping methods have been developed, including magnitude‐based methods, such as T_2_‐based methods [5, 6, 7, 8], calibrated fMRI [9, 10, 11, 12], and quantitative blood oxygen level dependent magnitude (qBOLD) [13, 14, 15], and phase‐based methods including quantitative susceptibility mapping (QSM) [16, 17, 18, 19].

Recently, an integrated model combining QSM and qBOLD (QQ) was introduced to consider OEF effects on both magnitude and phase signals in a multi‐echo gradient echo (mGRE) sequence [20]. QQ holds high clinical potential because it utilizes a single routine mGRE sequence available on clinical MRI scanners and eliminates the impractical vascular challenges required by other OEF methods [9]. QQ has been validated [21, 22, 23, 24] and clinically applied in neurological disorders [25, 26, 27, 28, 29, 30, 31, 32, 33, 34, 35, 36, 37, 38, 39, 40].

QQ robustness has been further improved using machine learning approaches [41, 42, 43, 44]. Notably, a deep learning approach, QQ‐NET [45], generates more accurate OEF maps with 150 times faster reconstruction than previous algorithms [42]. However, unlike its phase signal input (QSM), which is reconstructed before network input and unaffected by echo time (TE), QQ‐NET's magnitude signal input relies on fixed TE sampling and therefore requires identical TE sampling during training and testing. When acquisition protocols differ, the network must be retrained, limiting clinical deployment.

To address this limitation, we introduce QQ‐F, a novel deep learning model designed to generalize across diverse TE sampling schemes without retraining. QQ‐F incorporates a U‐Net architecture with a feature extraction unit (FEU) that derives signal‐, noise‐, and TE‐related features through exponential fitting. QQ‐F was compared with QQ‐NET in simulations and in multiple sclerosis (MS) and dementia patients with varying TE sets to assess robustness in clinical applications.

## 2 Methods

### 2.1 Data Acquisition

This study retrospectively analyzed datasets from our previous OEF studies approved by the Weill Cornell Medicine Institutional Review Board. The sample included 24 relapsing–remitting MS patients diagnosed by the McDonald criteria [46] with at least one T2FLAIR‐defined lesion (18 females, 6 males; mean age, 38 ± 8 years; scan time, 7 min per subject) and 30 cognitively impaired patients suspected to have AD based on neurological examination, neuropsychological assessment, laboratory testing, and MRI (14 females, 16 males; mean age, 72 ± 9 years; scan time, 4 min per subject) [31, 47]. All participants provided written informed consent in accordance with the ethical standards of the 1975 Helsinki Declaration and its later amendments.

### 2.2 Data Processing: QSM and CBF

QSM reconstruction was performed by estimating the total field via a nonlinear fit of the mGRE [48], obtaining the local field by the Projection onto Dipole Fields method [49], and computing susceptibility with the Morphology Enabled Dipole Inversion with automatic uniform cerebrospinal fluid zero reference algorithm [50, 51, 52, 53]. All images were co‐registered and interpolated to the resolution of the QSM maps using the FSL FLIRT algorithm [54, 55].

### 2.3 QQ Model

The QQ model combines QSM (phase signal analysis) and qBOLD (magnitude signal analysis) to estimate $\mathrm{OEF}=1-Y/Y_{a}$ with venous (Y) and arterial $\left(Y_{a}=0.98\right)$ oxygenation [20]. The QSM‐based model divides voxel‐wise susceptibility into contributions from venous blood (OEF effect) and nonblood neural tissue susceptibility ($\chi_{n}$):

(1) $$\begin{array}{cc} F_{\mathrm{QSM}}\left(Y,v,\chi_{n}\right) & =\left[\frac{\chi_{\mathrm{ba}}}{\alpha }+\psi_{\mathrm{Hb}}\cdot \Delta \chi_{\mathrm{Hb}}\cdot \left(-Y+\frac{1-\left(1-\alpha \right)\cdot Y_{a}}{\alpha }\right)\right]\\ & \;\cdot v+\left(1-\frac{v}{\alpha }\right)\cdot \chi_{n} \end{array}$$

where $\chi_{\mathrm{ba}}=-108.3\mathrm{ppb}$ is the fully oxygenated blood susceptibility assuming tissue hematocrit Hct = 0.357 [19], α=0.77 is the ratio between the venous blood volume (v) and total blood volume [56], $\psi_{\mathrm{Hb}}=0.0909$ is the hemoglobin volume fraction with Hct = 0.357 [17], $\Delta \chi_{\mathrm{Hb}}=12522\mathrm{ppb}$ is the susceptibility difference between deoxy‐ and oxyhemoglobin [57, 58].

The qBOLD models the OEF effect on the mGRE magnitude:

(2) $$\begin{array}{c} F_{\text{qBOLD}}\left(S_{0},R_{2},Y,v,\chi_{n},\mathrm{TE}\right)=S_{0}\cdot e^{-R_{2}\cdot \mathrm{TE}}\cdot F_{\text{BOLD}}\left(Y,v,\chi_{n},\mathrm{TE}\right)\cdot G\left(\mathrm{TE}\right) \end{array}$$

where $S_{0}$ is signal intensity at t=0, $R_{2}$ is the transverse relaxation rate, G(TE) is the macroscopic field effect estimated by the voxel spread function method [59], $F_{\text{BOLD}}=\exp\left[-vf_{s}\left(\delta \omega \cdot \mathrm{TE}\right)\right]$, having distinct asymptotic behaviors in the exponential term: quadratic at short TE and linear at long TE [60, 61]:

(3) $$\begin{array}{c} F_{\text{BOLD}}\left(Y,v,\chi_{n},\mathrm{TE}\right)=\left\{\begin{array}{ll} e^{-v\cdot \frac{3}{10}{\left(\delta \omega \cdot \mathrm{TE}\right)}^{2}}, & \;\mathrm{TE}<1.5t_{c}\\ e^{-v\cdot \left(\delta \omega \cdot \mathrm{TE}-1\right)}, & \;\mathrm{TE}>1.5t_{c} \end{array}\right. \end{array}$$

where $1.5t_{c}=1.5/\delta \omega$ is considered to be the boundary of two‐TE regimes. $\delta \omega =\frac{1}{3}\cdot \gamma \cdot B_{0}\cdot \left[\psi_{\mathrm{Hb}}\cdot \Delta \chi_{\mathrm{Hb}}\cdot \left(1-Y\right)+\chi_{\mathrm{ba}}-\chi_{n}\right]$ is the characteristic frequency due to the susceptibility difference between deoxygenated blood and the surrounding tissue [20].

### 2.4 QQ‐F: QQ‐NET With Feature Extraction Unit

QQ‐NET solves QQ (Equations 1 and 2) using a U‐Net trained on a fixed TE, requiring retraining when TE sampling differs [62]. To resolve this limitation, QQ‐F introduce a FEU (Figure 1) that extracts features from the mGRE magnitude signals using multi‐range exponential fitting:

(4) $$\begin{array}{cc} & F_{\text{qBOLD}}\left(S_{0},R_{2},Y,v,\chi_{n},\mathrm{TE}\right)/G\left(\mathrm{TE}\right)\\ & \;=\left\{\begin{array}{l} S_{0}\cdot e^{-R_{2}\cdot \mathrm{TE}}\cdot e^{-v\cdot \frac{3}{10}{\left(\delta \omega \cdot \mathrm{TE}\right)}^{2}},\;\mathrm{TE}<1.5t_{c}\\ S_{0}\cdot e^{-R_{2}\cdot \mathrm{TE}}\cdot e^{-v\cdot \left(\delta \omega \cdot \mathrm{TE}-1\right)},\;\mathrm{TE}>1.5t_{c} \end{array}\right. \end{array}$$

**FIGURE 1:**
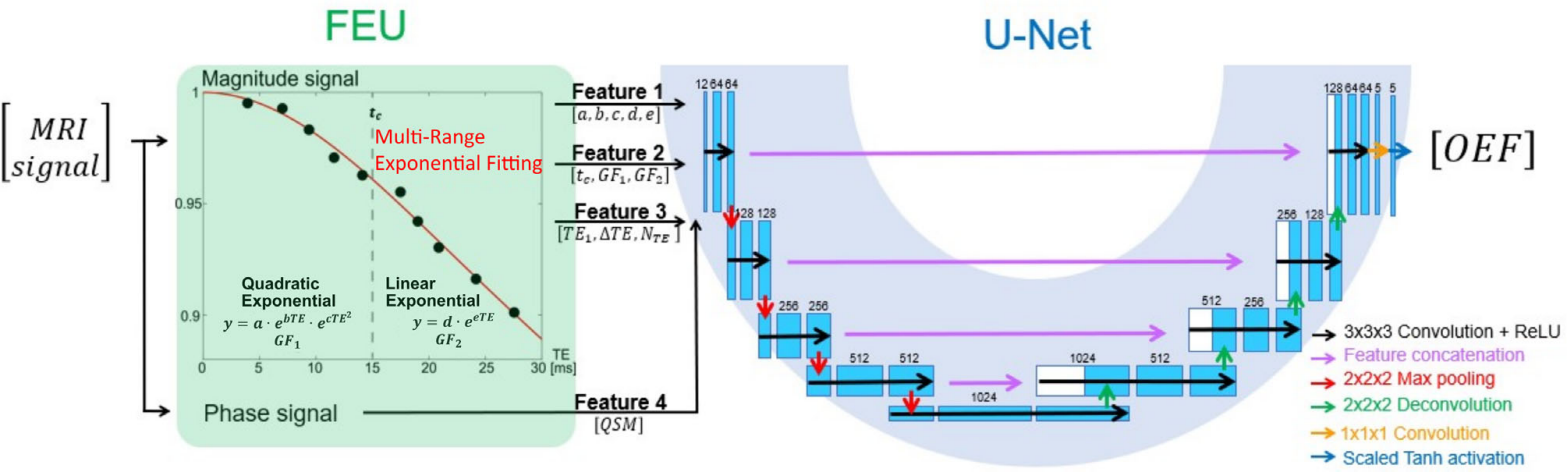
QQ‐F structure. QQ‐F consists of feature extraction unit (FEU) and U‐NET. FEU extract features from magnitude signal to capture signal characteristics and the residual of the fitting. These features are combined with QSM as input for the U‐Net which outputs model parameter.

This formulation can be expressed as a polynomial exponential function with five coefficients:

(5) $$\begin{array}{cc} & F_{\text{qBOLD}}\left(S_{0},R_{2},Y,v,\chi_{n},\mathrm{TE}\right)/G\left(\mathrm{TE}\right)\\ & \;=\left\{\begin{array}{l} a\cdot e^{\mathrm{bTE}}\cdot e^{c{\mathrm{TE}}^{2}},\;\mathrm{TE}<1.5t_{c}\\ d\cdot e^{\mathrm{eTE}},\;\mathrm{TE}>1.5t_{c} \end{array}\right. \end{array}$$

where $a=S_{0},b=-R_{2},c=-\frac{3}{10}v{\left(\delta \omega \right)}^{2},d=S_{0}e^{v},e=-\left(R_{2}+\right.\left.v\delta \omega \right)$. To minimize fitting error (e.g., from logarithmic transformation) and ensure mathematical stability and smooth transition between quadratic and linear regimes, we employed the Levenberg‐Marquardt (LM) method with regularization to enforce continuity of both signal intensity and its gradient at $t_{c}$ [63] (Note S1).

We selected four feature groups to provide a comprehensive, physically informed representation of the signal and its uncertainty. Feature 1 (magnitude coefficients derived from Equations 4 and 5) maps analytically to specific QQ parameters ($S_{0},R_{2},Y,v,\chi_{n}$), providing a physics‐informed foundation for QQ parameter estimation. Feature 2 (fitting residual) serves as a spatial uncertainty indicator by identifying voxels have less reliable coefficient estimates due to noise or poor signal sampling. Feature 3 (first echo time, echo spacing, and total echoes) provides the acquisition context, enabling the network to account for how the TE configuration affects coefficient estimation accuracy. Feature 4 (QSM derived from phase data) provides the phase‐domain information, leveraging its direct analytic relationship with QQ parameters (Equation 1). As QSM is TE‐independent, it is incorporated in the same way as in QQ‐NET. The FEU outputs form an 11‐channel input tensor: 5 channels of magnitude coefficients (Feature 1), 2 channels of fitting residual (Feature 2), 3 channels of TE scheme information broadcast spatially to match image dimensions (Feature 3), and 1 channel of QSM (Feature 4). The U‐Net takes randomly sampled patches (size [11, 200, 200, 48]) within a brain mask as input, following the QQ‐NET training scheme for a fair comparison [45].

For a fair comparison with QQ‐NET, QQ‐F was trained on the same 26 simulated stroke brain datasets (SNR 100) used to train QQ‐NET, with one exception: the datasets were generated using five clinically representative TE sequences (Table S1) [25, 31, 64]. In contrast, QQ‐NET used a single TE set (TE_1_/ΔTE/TE_8_ = 4.5/5.0/39.5 ms) [45]. Both models followed the same training strategy (see the QQ‐NET paper for detailed implementation) [45].

The two trained models were tested with three different datasets (Test Data 1, 2, and 3). *Test Data 1*: One additional simulated stroke brain constructed in the same way as the training datasets. These simulated data were tested using two TE sets: Case 1: the same TE set used for QQ‐NET training (TE_1_/ΔTE/TE_8_ = 4.5/5.0/39.5 ms) and Case 2: a different TE set (TE_1_/ΔTE/TE_8_ = 7.0/7.0/56.0 ms). This TE set was chosen to alter the sampling grid while ensuring at least two early echoes (< 15 ms) to adequately sample the quadratic signal decay regime. We used SNR 100 and repeated each test five times with different noise realizations to measure variability across trials. Additionally, to investigate the effect of noise‐level mismatch, we repeated test with SNR 100, 30, and 20, calculating the standard deviation across those trials. *Test Data 2*: 24 MS patients with a slightly different TE set from the QQ‐NET training TE set (TE_1_/ΔTE/TE_11_ = 4.5/4.8/52.5 ms, voxel size = 0.47 × 0.47 × 3.0 mm^3^). *Test Data 3*: 30 dementia patients with significantly different TE set from the QQ‐NET training TE set (TE_1_/ΔTE/TE_10_ = 6.7/4.1/43.6 ms, voxel size = 0.75 × 0.75 × 3.0 mm^3^). For Test data 2 and 3, QQ‐NET used the first eight echoes as input due to its requirement for a fixed‐size input, whereas QQ‐F was unaffected by changes in the TE sets.

### 2.5 Statistical Analysis

In the simulation (Test Data 1), accuracy and precision were calculated by mean absolute error ($\mathrm{MAE}\equiv \frac{1}{N_{v}}\sum_{i=1}^{N_{v}}\left|\mathrm{OE}F_{\text{truth}}-\mathrm{OE}F_{\mathrm{avg}}\right|$) and mean standard deviation (MSD $\equiv \frac{1}{N_{v}}\sum_{i=1}^{N_{v}}\mathrm{OE}F_{\mathrm{std}}$) where $\mathrm{OE}F_{\mathrm{avg}}\equiv \frac{1}{N_{t}}\sum_{j=1}^{N_{t}}\mathrm{OE}F_{i,j}$, $\mathrm{OE}F_{\mathrm{std}}\equiv \sqrt{\frac{1}{N_{t}}\sum_{j=1}^{N_{t}}{\left(\mathrm{OE}F_{i,j}-\frac{1}{N_{t}}\sum_{j=1}^{N_{t}}\mathrm{OE}F_{i,j}\right)}^{2}}$, i: the voxel index, j: the trial index, $N_{v}$: the number of voxels, $N_{t}$: the number of trials.

For MS data (Test Data 2) lesion‐specific analysis, two neuroradiologists (S.Z., 10 years of experience and W.H., 15 years of experience) identified lesions on T2FLAIR based on their relative contrast with respect to the adjacent normal appearing white matter [31]. A total of 485 were identified across 24 patients (median, 16 per patient; range: 6–46). For dementia data (Test Data 3) lesion‐specific analysis, a neuroradiologist (G.C., 11 years of experience) identified white matter hyperintensities (WMH) lesions which is associated with metabolic abnormalities and disease progression in dementia [25, 47]. Across 30 patients, 394 lesions were identified (median, 9 per patient; range: 1–53).

The detectability of lesion OEF abnormalities in the two real datasets was quantified using the contrast‐to‐noise ratio between the lesion and its adjacent normal tissue ($\mathrm{CNR}\equiv \frac{\left|\bar{\mathrm{OE}F_{\text{lesion}}}-\bar{\mathrm{OE}F_{\text{normal tissue}}}\right|}{\sigma \left({\mathrm{OEF}}_{\text{normal tissue}}\right)}$). The adjacent normal tissue was defined by dilating the lesion by three voxels and then subtracting the original lesion mask from the dilated mask (Figure S1). CNR, OEF average, OEF standard deviation (defined here as the spatial variation of voxel‐wise OEF values within each ROI) were compared between QQ‐F and QQ‐NET using a linear mixed‐effects model (LMM). For MS data, sex, age, disease duration, expanded disability status scale, and model (QQ‐F vs. QQ‐NET) were included as fixed effects. For dementia data, sex, age, Montreal cognitive assessment and model (QQ‐F vs. QQ‐NET) were included as fixed effects. For both datasets, subject was treated as a random effect to account for within‐subject correlations. A *p* value less than 0.05 was considered significant.

## 3 Results

Figure 2 compares OEF maps between QQ‐F and QQ‐NET using simulated data (Test Data 1). When tested with the QQ‐NET training TE set, the two models showed similar accuracy (MAE: QQ‐F vs. QQ‐NET: 3.99% vs. 4.93%). In contrast, when tested with a different TE set, QQ‐F provided significantly higher accuracy (MAE: 3.93% vs. 6.68%), successfully capturing low lesion OEF abnormalities that QQ‐NET failed to depict. Both models yielded small MSD values across five noise realizations (MSD: 0.30% vs. 0.37% in Case 1 and 0.15% vs. 0.47% in Case 2).

**FIGURE 2:**
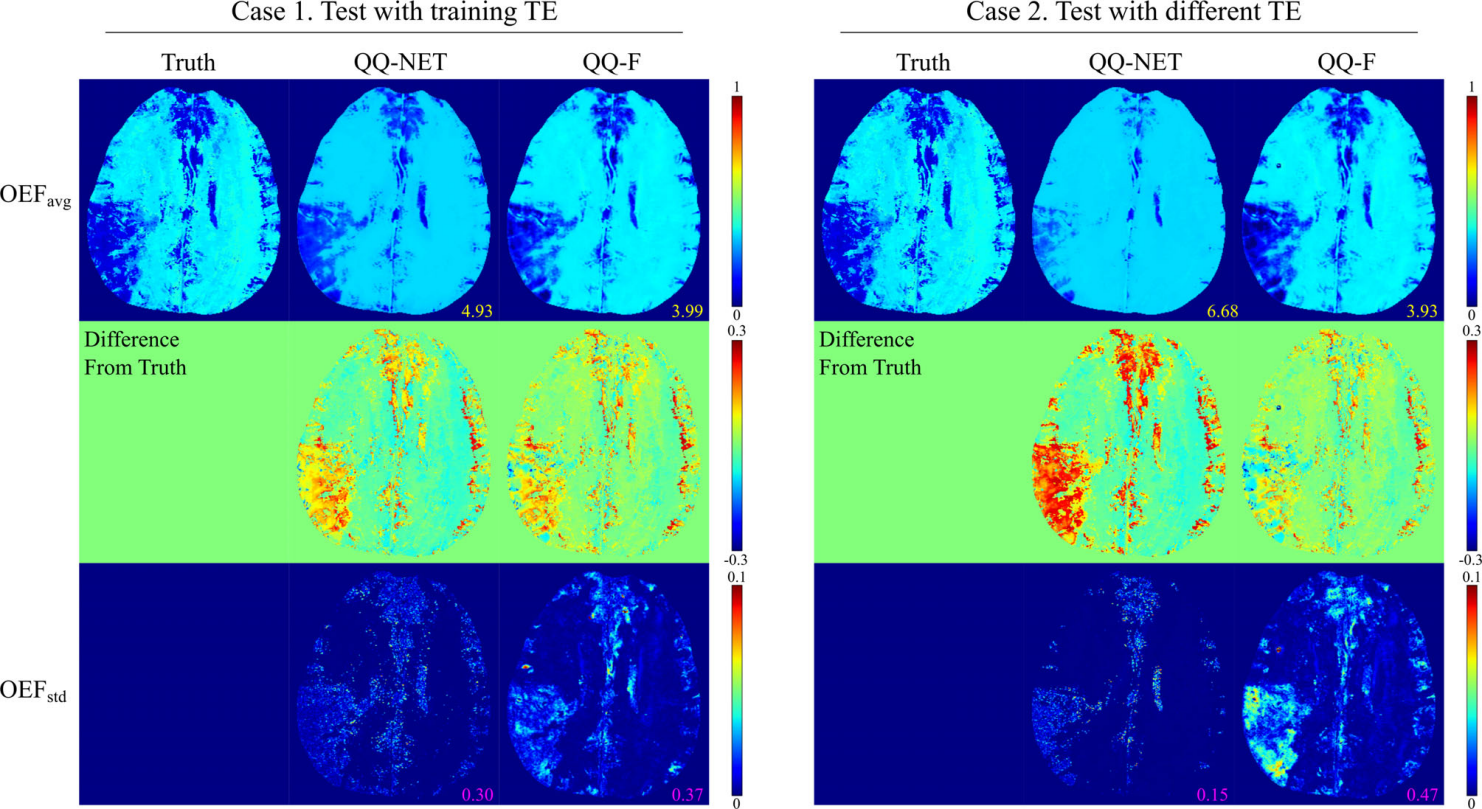
Comparison of OEF mapping between QQ‐NET and QQ‐F in simulated data. Compared to QQ‐NET, QQ‐F showed similar OEF when tested with the same TE as the training TE (MAE: 3.99% vs. 4.93%) and provided substantially more accurate OEF when tested with a different TE than the training TE (MAE: 3.93% vs. 6.68%). Both models were robust to noise (MSD: 0.30% vs. 0.37% in Case 1 and 0.15% vs. 0.47% in Case 2). OEF_avg_ and OEF_std_ indicate the average and SD OEF maps, respectively, among five trials. OEF_avg_ and OEF_std_ are shown in the unit of [%].

Figure 3 shows representative OEF maps from MS patients (Test Data 2). When the test TE set differed slightly from QQ‐NET training, both models provided uniform OEF in healthy tissue, as expected. However, QQ‐NET provided lower healthy‐tissue OEF than QQ‐F (e.g., 24.33% ± 3.81% vs. 27.42% ± 3.58%, *p* < 0.001). For lesion detection, QQ‐F showed improved spatial overlap between low‐OEF regions and MS lesions.

**FIGURE 3:**
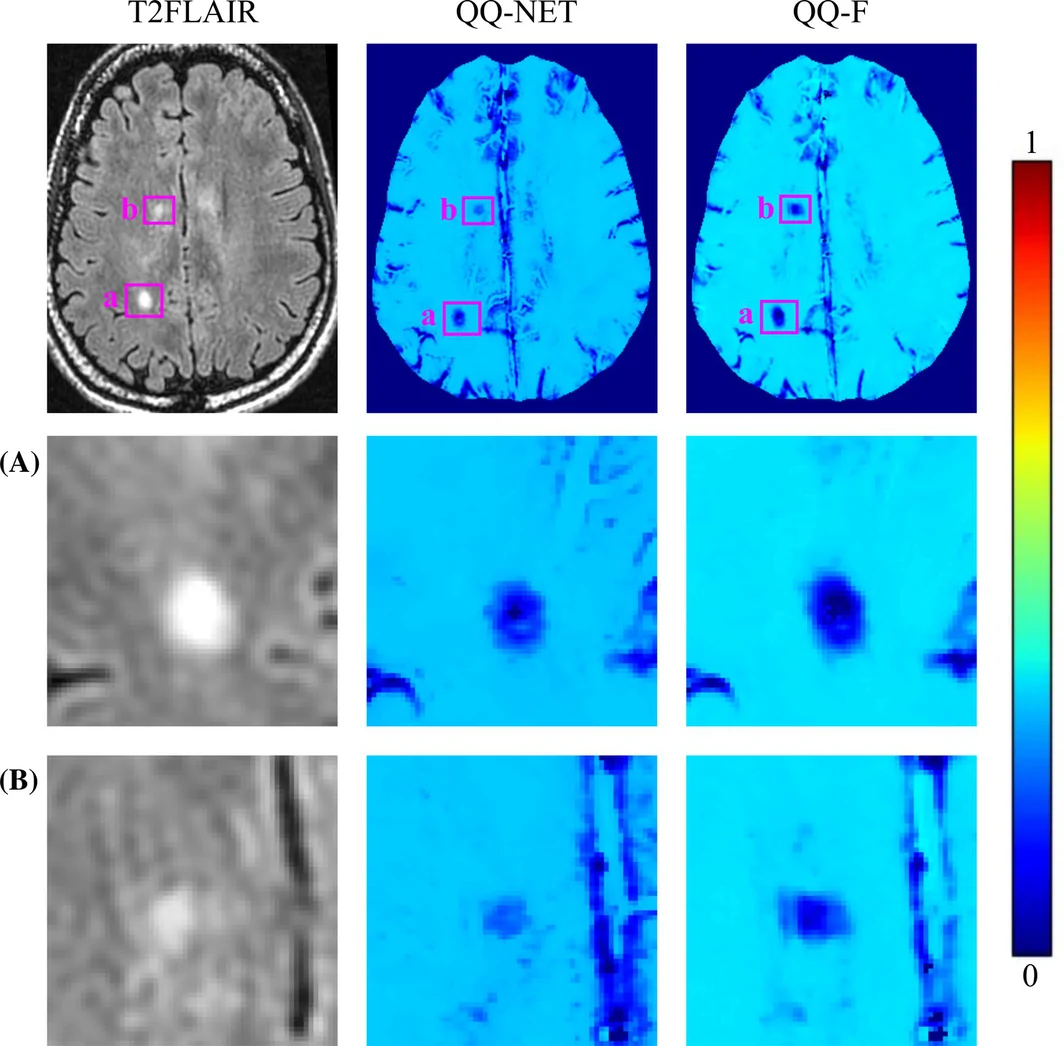
Comparison of OEF mapping between QQ‐NET and QQ‐F in a multiple sclerosis (MS) dataset showing two lesions. Both QQ‐F and QQ‐NET showed uniform OEF maps. However, the low OEF regions identified by QQ‐F showed substantial overlap with the T2FLAIR‐defined lesions, whereas QQ‐NET did not clearly depict the lesions in the OEF maps.

Figure 4 shows representative OEF maps from dementia patients (Test Data 3). When the test TE set differed significantly from QQ‐NET training, QQ‐F provided uniform OEF in healthy tissue, whereas QQ‐NET provided nonuniform and abnormally low OEF values, inconsistent with previous studies (QQ‐NET: 18.25% ± 4.27%; QQ‐F: 24.67% ± 4.13%; previous study: 24.84% ± 5.42% [47]). QQ‐F also depicted lesion boundaries more clearly than QQ‐NET.

**FIGURE 4:**
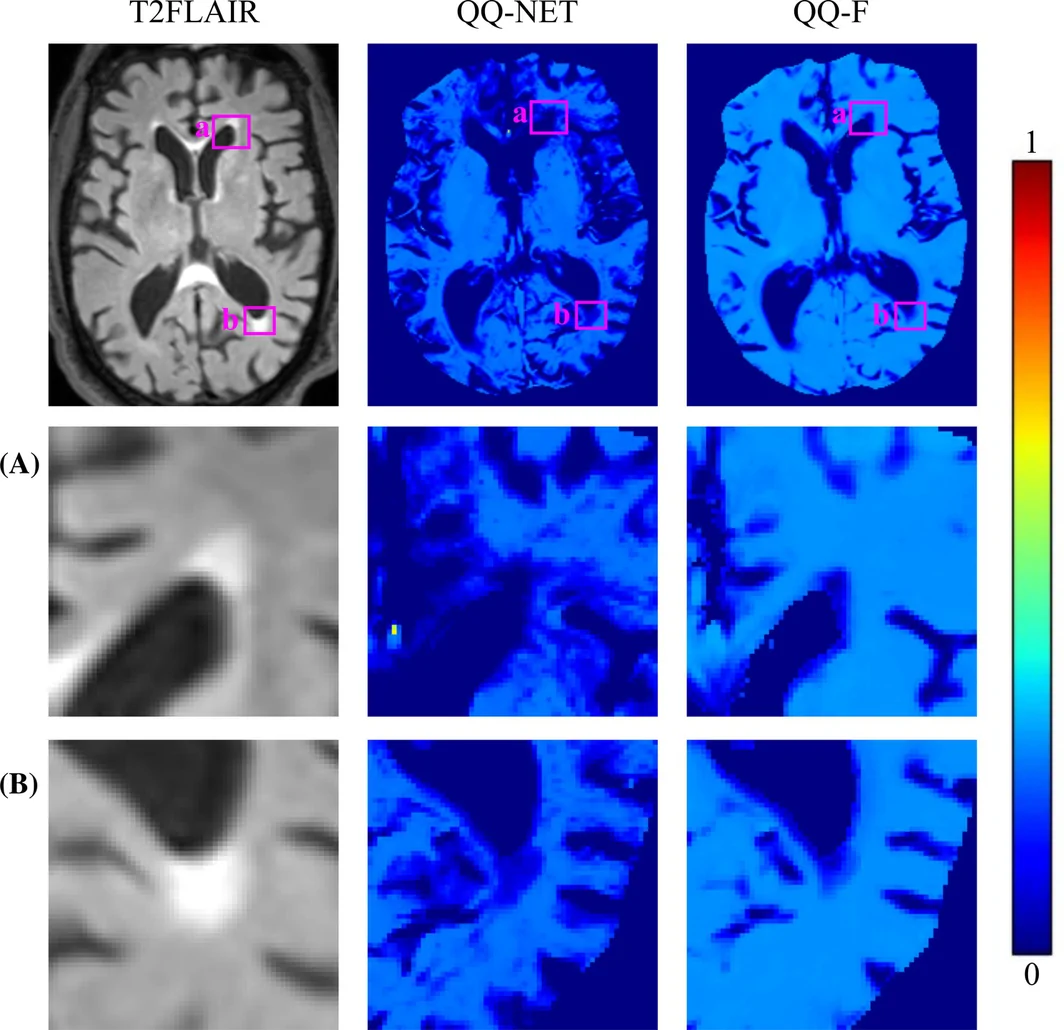
Comparison of OEF mapping between QQ‐NET and QQ‐F in dementia dataset showing four lesions. QQ‐F provided uniform OEF maps in healthy tissue, whereas QQ‐NET provided inaccurate and abnormally low OEF maps in gray matter. Besides, QQ‐F clearly depicted the boundary of lesions identified by T2FLAIR whereas QQ‐NET did not clearly depict boundary.

Figure 5 summarizes lesion‐to‐normal OEF CNR (Figure 5A), average OEF values in lesions and their adjacent normal tissue (Figure 5B), and OEF standard deviation in MS and dementia patients (Figure 5C). QQ‐F provided significantly higher CNR than QQ‐NET in both MS (1.04 ± 0.47 vs. 1.20 ± 0.58, *p* < 0.001) and dementia (0.29 ± 0.24 vs. 0.57 ± 0.39, *p* = 0.004). Both models showed significantly lower lesion OEF than adjacent normal tissue in MS and dementia. QQ‐F yielded lower OEF standard deviation than QQ‐NET in lesions (11.83% ± 1.84% vs. 8.73% ± 1.17%, *p* = 0.003) and adjacent normal‐appearing tissue (10.84% ± 1.40% vs. 7.74% ± 2.10%, *p* < 0.001) for dementia patients, but showed no significant difference in MS patients (5.62% ± 1.83% vs. 5.77% ± 2.06%, *p* = 0.50 in lesions or 4.67% ± 1.44% vs. 4.85% ± 1.70%, *p* = 0.43 in adjacent normal‐appearing tissue).

**FIGURE 5:**
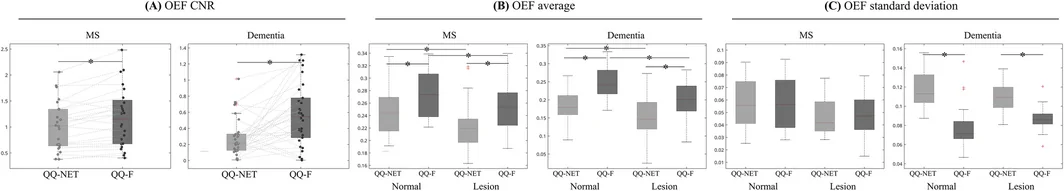
Box plots of OEF CNR between the lesion and its adjacent normal tissue (A), OEF average in the lesion and the normal tissue (B), and OEF SD in the normal tissue (C) in multiple sclerosis (MS) and dementia patients (Test Data 2 and 3). Red line, box, black whisker, and red cross indicate median, interquartile range, the range extending to 1.5 of the interquartile range, and outliers beyond the whisker range, respectively. Asterisk (*) indicates a significant difference (*p* < 0.05).

## 4 Discussion

This study demonstrates the feasibility of QQ‐F, a deep learning approach for OEF mapping that can process routine MRI datasets acquired with various sampling schemes without retraining. Compared with the existing QQ‐NET, QQ‐F showed improved accuracy in simulations (Figure 2) and higher lesion‐to‐normal tissue contrast in real datasets (Figures 3, 4, 5), particularly when TE schemes significantly differed from those used during QQ‐NET training. The primary limitation of QQ‐NET is its dependence on a fixed TE sampling scheme. When test data differ from the training protocol, the network requires echo padding or truncation, which may discard information and reduce generalizability (e.g., leading to systematic errors in OEF estimation, as shown in Figures 1 and 4). QQ‐F addresses this limitation by introducing a FEU that derives model‐related features through nonlinear fitting rather than directly using raw magnitude signals. This allows the network to process data acquired with diverse TE schemes while preserving signal characteristics. Furthermore, because this voxel‐wise feature extraction provides physics‐informed model input, QQ‐F is expected to generalize robustly across lesion sizes, enabling characterization of small in vivo pathologies despite training on larger simulated hypoxic regions. Hence, QQ‐F enables robust QQ‐based OEF mapping across diverse acquisition protocols using a standard mGRE sequence.

The FEU is central to QQ‐F's generalization capability because its reliability directly affects the entire framework. We therefore evaluated FEU reliability across feature design, fitting stability, noise sensitivity, and TE variability. For feature design, the four feature groups were selected to provide a comprehensive, physics‐informed representation of the signal and its uncertainty. An ablation analysis showed that removing Feature 3 (TE scheme information) degraded performance on unseen TE schemes (Table S2), confirming the importance of explicit acquisition context for protocol generalizability. For fitting stability, Levenberg‐Marquardt fitting with continuity regularization produced a failure rate below 0.01% across 59 million voxels at SNR 20 (Figure S6), and the fitting residual (Feature 2) was forwarded to the U‐Net as a spatial uncertainty indicator to mitigate localized instabilities. For noise sensitivity, a perturbation analysis showed coefficient deviations below 9% at SNR 20 (Table S3). This input‐level perturbation did not meaningfully degrade final OEF estimation. End‐to‐end MAE increased by only 0.24% from SNR 100 to SNR 20, comparable to the 0.11% increase in QQ‐NET without FEU, indicating that fitting does not amplify noise beyond the network's inherent noise sensitivity (Figure S2). For TE variability, changes in TE_1_ or ΔTE produced no systematic MAE change, indicating robustness within clinical TE ranges. Reducing the number of echoes moderately increased MAE, as expected from reduced fitting overdetermination, but performance remained acceptable even with five echoes (Table S4 and Figure S4).

In simulations, QQ‐F provided lower MAE and better lesion detectability, particularly when tested with a TE set different from training (Figure 2). QQ‐NET's higher MAE may reflect TE discrepancies between training and testing, whereas QQ‐F remained robust by using TE‐independent features. OEF variability across trials in QQ‐F (OEF_std_) may arise from fitting errors during feature extraction that potentially propagate through subsequent network layers (Figure 1) but does not meaningfully affect overall prediction performance. When SNR decreased from 100 to 20, the MAE increase was comparable to the noise propagation level in QQ‐NET [45] (Figure S2). Furthermore, in the dementia datasets, QQ‐F produced a lower standard deviation than QQ‐NET in normal tissue (Figure 5C), consistent with the uniform OEF distribution expected from ^15^O PET. The higher QQ‐NET standard deviation in dementia normal tissue may reflect inaccurate OEF predictions, particularly in gray matter (Figure 4), making lesions harder to distinguish from surrounding normal tissue.

In the MS datasets (Figure 3), where the TE set slightly differed from QQ‐NET's training (TE_1_/ΔTE/TE_10_ = 6.7/4.1/43.2 vs. TE_1_/ΔTE/TE_8_ = 4.5/5/39.5 ms), both models provided uniform OEF maps in nonlesional healthy tissue. Both models also showed lower lesion OEF than healthy tissue (Figure 5B), consistent with irreversible tissue damage reported in MS histopathology studies [65, 66, 67]. QQ‐F provided significantly higher CNR than QQ‐NET (Figure 5A) and more clearly delineated lesion boundaries in OEF maps (Figure 3). This likely reflects QQ‐NET's sensitivity to the slight TE mismatch (ΔTE = 4.8 vs. 5.0 ms): Because QQ‐NET takes raw magnitude signals as input, its performance is sensitive to deviations in echo spacing from training. In contrast, QQ‐F's FEU extracts physics‐informed features that explicitly account for the TE scheme, making it inherently robust to acquisition differences.

In the dementia dataset (Figure 4), where the TE set significantly differed from QQ‐NET's training (TE_1_/ΔTE/TE_10_ = 6.7/4.1/43.2 vs. TE_1_/ΔTE/TE_8_ = 4.5/5/39.5 ms), QQ‐F yielded uniform healthy‐tissue OEF and clear lesion boundaries (Figure 4), whereas QQ‐NET provided nonuniform OEF maps with abnormally low values. This is consistent with QQ‐F achieving higher OEF CNR and lower standard deviation (Figure 5A,C). Both models also provided lower OEF in WMH than in normal tissue (Figure 5B), consistent with decreased WMH OEF reported in dementia [47], suggesting tissue damages within WMH. Although early hypoperfusion may elevate OEF in surrounding at‐risk watershed regions [68], reduced OEF in mature WMH cores likely reflects late‐stage structural and capillary damage that impairs oxygen extraction [69]. This is further supported by QQ‐F's lower WMH venous blood volume (v) (Figure S3).

To determine whether QQ‐F's superior CNR reflected its use of additional echoes relative to QQ‐NET rather than architectural advantage, we repeated the analysis using only the same 8 echoes as QQ‐NET. QQ‐F CNR was essentially unchanged (MS: 1.20 ± 0.58 vs. 1.23 ± 0.63; dementia: 0.57 ± 0.34 vs. 0.57 ± 0.39) and still significantly outperformed QQ‐NET under matched input conditions (MS: *p* < 0.001; dementia: *p* = 0.001) (Figure S5). This 8‐echo restriction is not an artificial constraint but reflects QQ‐NET's inherent limitation with nonmatching TE schemes, which QQ‐F is designed to overcome.

The abnormally low OEF values in normal tissue from QQ‐NET, especially in dementia (Figures 4 and 5B), suggest that QQ‐NET's ability to capture OEF distribution may be compromised by TE discrepancies. Since signal magnitude decreases monotonically, a test sequence with longer TEs than training yields systematically lower signal intensities at each sampling point. Because QQ‐NET maps raw magnitude signals directly to OEF based on its training scheme, it likely misinterprets this acquisition‐driven signal reduction as a physiological decrease in OEF. In contrast, QQ‐F extracts high‐level features from the signal rather than relying on raw magnitudes, making it more robust to TE variations and, therefore, more suitable for clinical applications where protocol differences are common.

Despite its improved generalizability, QQ‐F has several limitations. First, like QQ‐NET, it requires prior information on the model parameters $\left(S_{0},R_{2},Y,v,\chi_{n}\right)$ [45]. Although QQ‐F training covered a wide physiological range for the model parameters, including the full OEF range (0% – 100%) and v value range of 0% – 8% (which includes PET and MRI literature ranges of 0.5% – 5.5%) [70], real physiology may involve parameter combinations not fully represented in training. Second, training on 26 simulated stroke datasets introduces a domain gap with real‐world clinical data. These datasets were generated from in vivo scans processed with QQ‐CCTV to synthesize forward signals [43, 45]. This provides realistic parameter distributions but relies on QQ‐CCTV estimations as ground truth, which may propagate algorithmic biases. The stroke‐only source data may also limit generalizability to other pathologies. Future work should assess whether more diverse or disease‐matched training datasets improve in vivo performance. Third, QQ‐F may depend on the FEU fitting process. Although normal‐tissue MSD was acceptably small in simulations (Figure 2) and lower than that of a previous machine learning approach [43], the FEU may still introduce noise that propagates through subsequent model stages. Therefore, more advanced feature extraction methods may further improve performance and reduce noise propagation. Fourth, the 3.0 mm slice thickness in both MS and dementia datasets may introduce through‐plane partial volume effects, particularly in smaller lesions, and the impact of spatial resolution on QQ‐F performance warrants future investigation.

In conclusion, by introducing a FEU, QQ‐F enables deep learning‐based OEF mapping across diverse MRI sampling schemes without the need for retraining. This enhances the clinical scalability of the QQ framework.

## Funding

This work was supported by the National Institutes of Health (R00NS123229 and R01NS136369).

## Conflicts of Interest

The authors declare no conflicts of interest.

## Supporting information


**Table S1:** TE sequences used in QQ‐F training.
**Table S2:** OEF mean absolute error (MAE) on a TE sequence different from the training set with simplified feature input.
**Table S3:** Relative error of the 5 fitting coefficients compared to SNR 100 baseline.
**Table S4:** OEF MAE [%] of different TE sequence compared to baseline under SNR 100.
**Figure S1:** Examples of determining normal tissue. Lesion mask is the inner area of the red line. The normal tissue mask is determined by dilating the lesion area, which is shown by the red area.
**Figure S2:** Comparison between the OEFs obtained by QQ‐F at different SNRs (SNR 100, 30, and 20) in simulated data. Numbers indicate MAE (yellow) and MSD (pink). ${\mathrm{OEF}}_{\mathrm{avg}},{\mathrm{OEF}}_{\mathrm{std}}$ are shown in the unit of [%].
**Figure S3:** Comparison of OEF, v, $R_{2},$ and $\chi_{n}$ maps between QQ‐F and QQ‐NET in a multiple sclerosis patient (Test Data 3). QQ‐F provided low v values in the T2FLAIR defined lesion (purple arrow).
**Figure S4:** Comparison between the OEFs obtained by QQ‐F using different TE sampling strategies in simulated data under SNR 100. Baseline: TE_1_/ΔTE/TE_8_ = 4.5/5.0/39.5 ms; S1: change only TE1 to 7 ms, TE_1_/ΔTE/TE_8_ = 7.0/5.0/42.0 ms; S2: change only ΔTE to 7 ms, TE_1_/ΔTE/TE_8_ = 4.5/7.0/53.5 ms; S3: change both TE1 and ΔTE to 7 ms, TE_1_/ΔTE/TE_8_ = 7.0/7.0/56.0 ms; S4: reduce number of echoes to 5, TE_1_/ΔTE/TE_5_ = 4.5/5.0/24.5 ms. Numbers indicate MAE (yellow). ${\mathrm{OEF}}_{\mathrm{avg}}$ is shown in the unit of [%].
**Figure S5:** Box plots of OEF CNR between the lesion and its adjacent normal tissue between QQ‐NET and QQ‐F when restricting QQ‐F to use the same 8 echoes as QQ‐NET on multiple sclerosis (MS) and dementia patients (Test Data 2 and 3). Red line, box, black whisker, and red cross indicate median, interquartile range, the range extending to 1.5 of the interquartile range, and outliers beyond the whisker range, respectively. Asterisk (*) indicates a significant difference (*p* < 0.05).
**Figure S6:** Logarithmic distribution of 5 coefficients and physical boundaries under SNR20. The out‐of‐bound rates are smaller than 0.01% for all coefficients.
**Note S1:** The Levenberg‐Marquardt (LM) fitting loss function.

## Data Availability

The data that support the findings of this study are available from the corresponding author upon reasonable request.
