# Supplementary material for: A Generalized Approach to Solving Deep Learning‐Based Quantitative Susceptibility Mapping and Quantitative Blood Oxygen Level Dependent Magnitude (QSM + qBOLD or QQ) for Oxygen Extraction Fraction (OEF) Mapping Across Diverse Acquisition Schemes
Source: Magn Reson Med. 2026 Aug 4;96(6):2812–21. doi: 10.1002/mrm.70524 (PMC13619847; doi:10.1002/mrm.70524)
Supplement: Supplementary file 1 [file MRM-96-2812-s001.docx]

**Supporting information Table S1.** TE sequences used in QQ-F training

|  | Training TE sets |
| --- | --- |
| 1 | TE_1_/ΔTE/TE_11_ = 4.5/4.8/52.5 ms |
| 2 | TE_1_/ΔTE/TE_7_ = 2.3/3.9/25.7 ms |
| 3 | TE_1_/ΔTE/TE_10_ = 6.7/4.1/43.6 ms |
| 4 | TE_1_/ΔTE/TE_8_ = 4.5/4.9/38.8 ms |
| 5 | TE_1_/ΔTE/TE_6_ = 3.5/3.5/21 ms |

**Supporting information Table S2.** OEF mean absolute error (MAE) on a TE sequence different from the training set with simplified feature input.

| Model | SNR100 | SNR30 | SNR20 |
| --- | --- | --- | --- |
| Without Feature 3 | 3.72% | 3.76% | 4.27% |
| With Feature 3 | 3.58% | 3.76% | 3.93% |

**Supporting information Table S3.** Relative error of the 5 fitting coefficients compared to SNR 100 baseline

| Noise level | Coeff 1 | Coeff 2 | Coeff 3 | Coeff 4 | Coeff 5 |
| --- | --- | --- | --- | --- | --- |
| SNR50 | 1.43% | 3.25% | 2.94% | 1.43% | 3.25% |
| SNR30 | 2.21% | 5.04% | 4.82% | 2.21% | 5.04% |
| SNR20 | 3.23% | 7.37% | 8.88% | 3.23% | 7.38% |

**Supporting information Table S4**: OEF MAE [%] of different TE sequence compared to baseline under SNR 100

| ***Condition*** | ***TE1(ms)*** | $\boldsymbol{\Delta}$***TE (ms)*** | ***# of echoes*** | ***OEF MAE [%]*** |
| --- | --- | --- | --- | --- |
| *Baseline (training TE)* | *4.5* | *5.0* | *8* | *3.99* |
| *S1: TE1 change only* | ***7.0*** | *5.0* | *8* | *3.93* |
| *S2:* $\Delta$*TE change only* | *4.5* | ***7.0*** | *8* | *3.96* |
| *S3: TE1* $\Delta$*TE change* | ***7.0*** | ***7.0*** | *8* | *3.93* |
| *S4: Echo reduction only* | *4.5* | *5.0* | ***5*** | *4.15* |

**Supporting Information Figure S1.** Examples of determining normal tissue. Lesion mask is the inner area of the red line. The normal tissue mask is determined by dilating the lesion area, which is shown by the red area.

**Supporting Information Figure S2.** Comparison between the OEFs obtained by QQ-F at different SNRs (SNR 100, 30 and 20) in simulated data. Numbers indicate MAE (yellow) and MSD (pink). ${OEF}_{avg},{OEF}_{std}$ are shown in the unit of [%].

**Supporting Information Figure S3.** Comparison of OEF, $v$, $R_{2}$ and $\chi_{n}$ maps between QQ-F and QQ-NET in a multiple sclerosis patient (Test Data 3). QQ-F provided low $v$ values in the T2FLAIR defined lesion (purple arrow).

**Supporting Information Figure S4.** Comparison between the OEFs obtained by QQ-F using different TE sampling strategies in simulated data under SNR 100. Baseline: TE_1_/ΔTE/TE_8_ = 4.5/5.0/39.5 ms; S1: change only TE1 to 7ms, TE_1_/ΔTE/TE_8_ = 7.0/5.0/42.0 ms; S2: change only ΔTE to 7ms, TE_1_/ΔTE/TE_8_ = 4.5/7.0/53.5 ms; S3: change both TE1 and $\Delta$TE to 7ms, TE_1_/ΔTE/TE_8_ = 7.0/7.0/56.0 ms; S4: reduce number of echoes to 5, TE_1_/ΔTE/TE_5_ = 4.5/5.0/24.5 ms. Numbers indicate MAE (yellow). ${OEF}_{avg}$ is shown in the unit of [%].
**Supporting Information Figure S5** Box plots of OEF CNR between the lesion and its adjacent normal tissue between QQ-NET and QQ-F when restricting QQ-F to use the same 8 echoes as QQ-NET on multiple sclerosis (MS) and dementia patients (Test Data 2 and 3). Red line, blue box, black whisker, and red cross indicate median, interquartile range, the range extending to 1.5 of the interquartile range, and outliers beyond the whisker range, respectively. Asterisk (*) indicates a significant difference (p < 0.05).

**Supporting Information Figure S6.** Logarithmic distribution of 5 coefficients and physical boundaries under SNR20. The out-of-bound rates are smaller than 0.01% for all coefficients.

**Supporting Information Note S1.** The Levenberg-Marquardt (LM) fitting Loss Function

At the fitting level, we incorporate regularization directly into the Levenberg-Marquardt (LM) fitting algorithm itself to guarantee mathematical stability. The loss function is formulated as:

$$Loss=\lambda_{1}\cdot\left( M-\hat{M} \right)^{2}+\lambda_{2}\cdot\left( V_{quad}-V_{lin} \right)^{2}+\lambda_{3}\cdot\left( D_{quad}-D_{lin} \right)^{2}$$

where *M* is the raw magnitude signal, $\hat{M}$ is the estimated magnitude signal. The terms $V_{quad}$ and $V_{lin}$ represent the evaluated values of the quadratic and linear fitted curves at the fitting boundary (TE = 1.5tc), while $D_{quad}$ and $D_{lin}$ represent their respective slopes (derivatives) at this boundary. The regularization weights $\lambda_{1}$, $\lambda_{2}$, $\lambda_{3}$ are set to 1, 1e3, 1e3 respectively.
